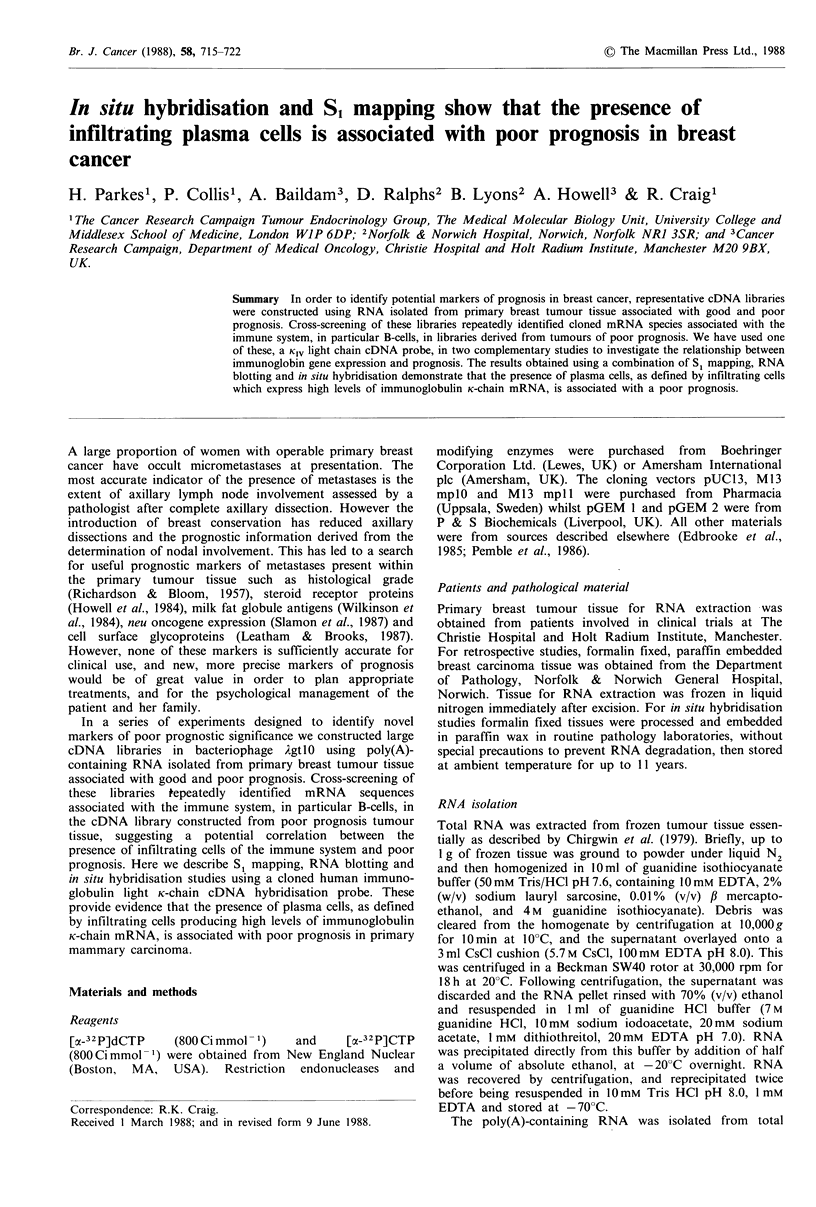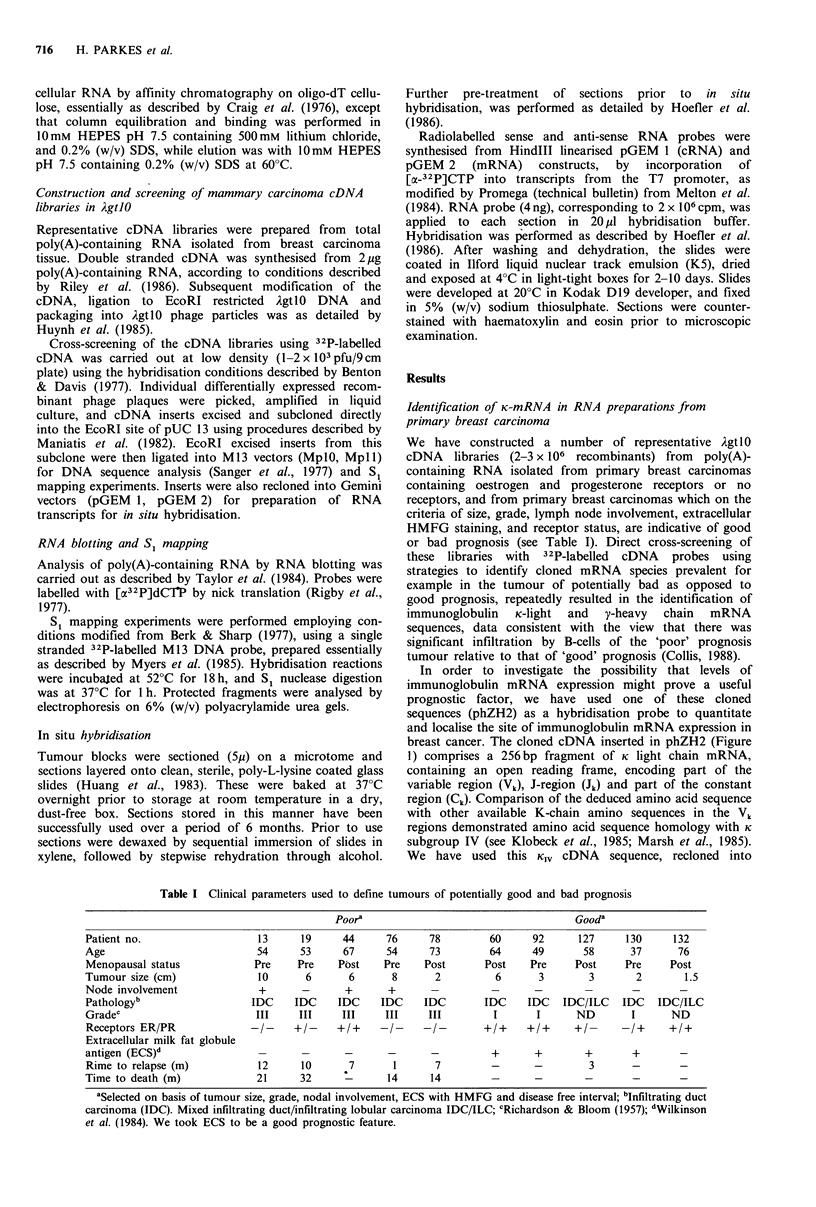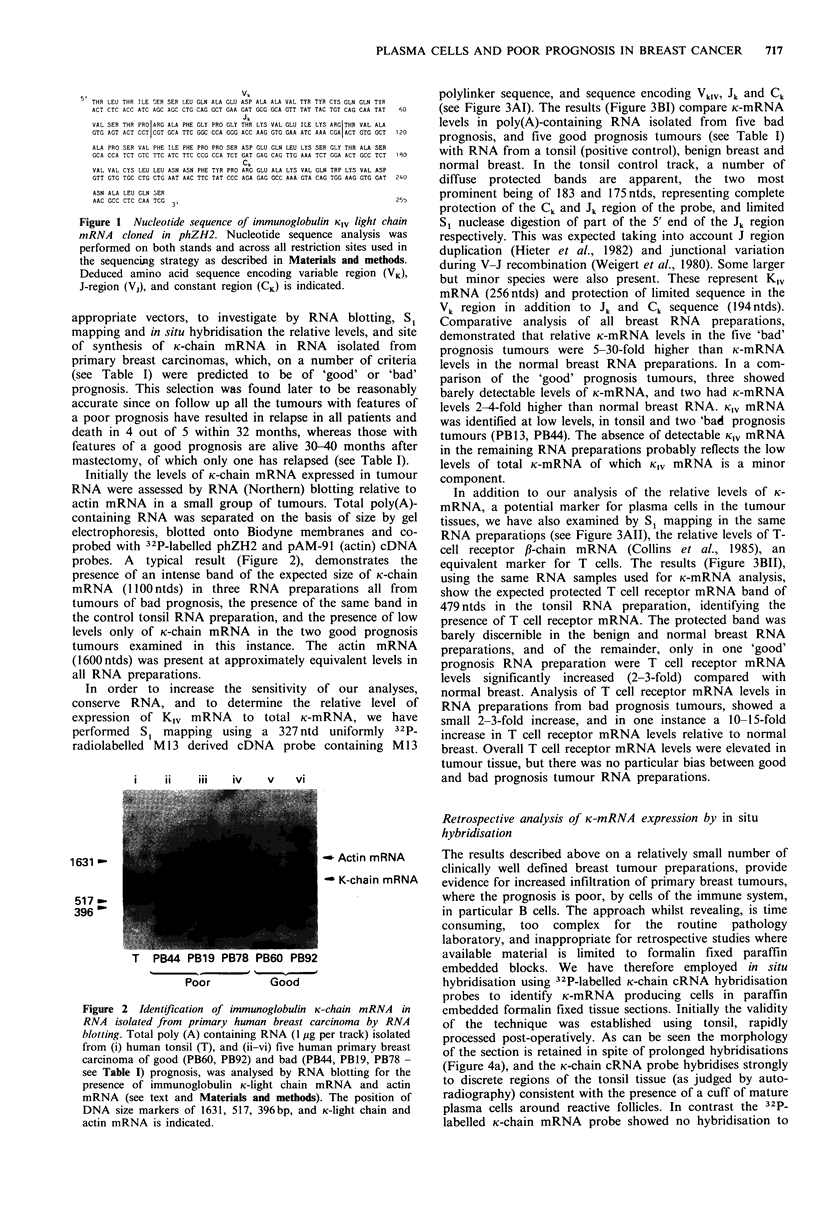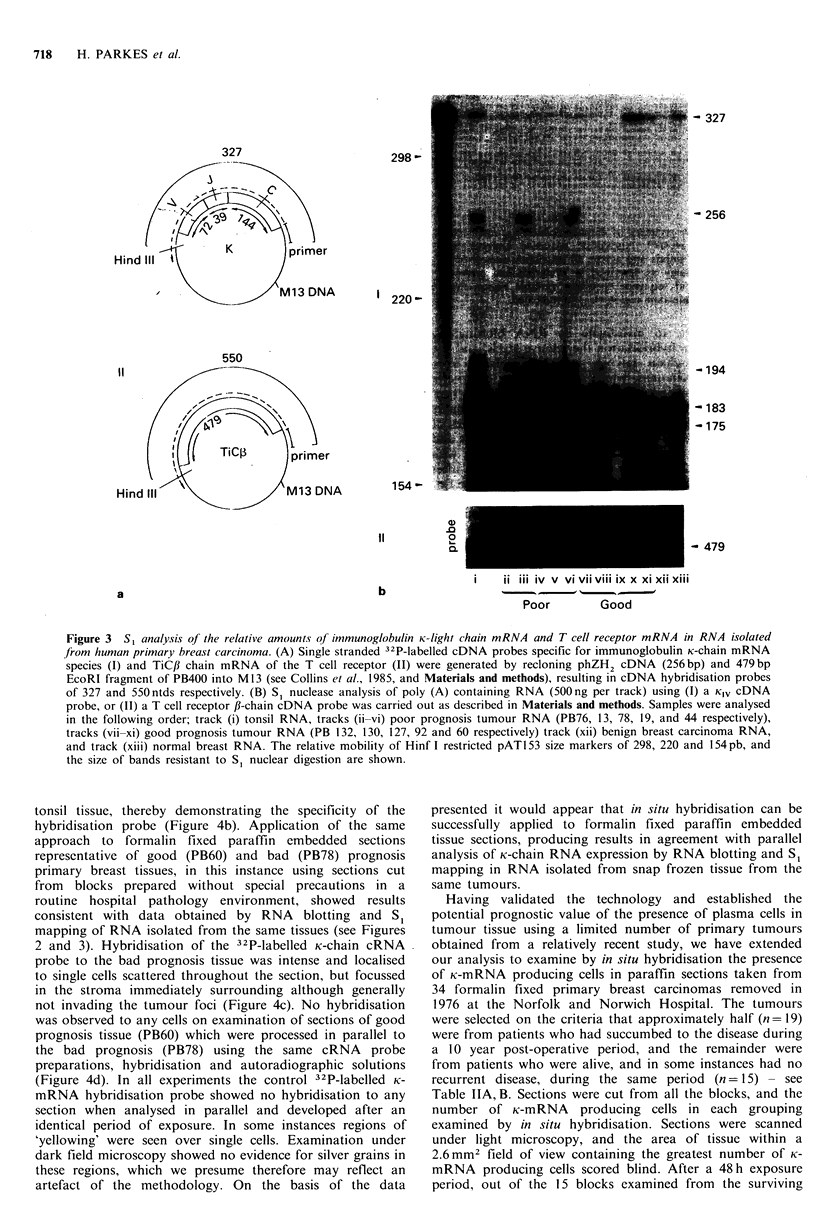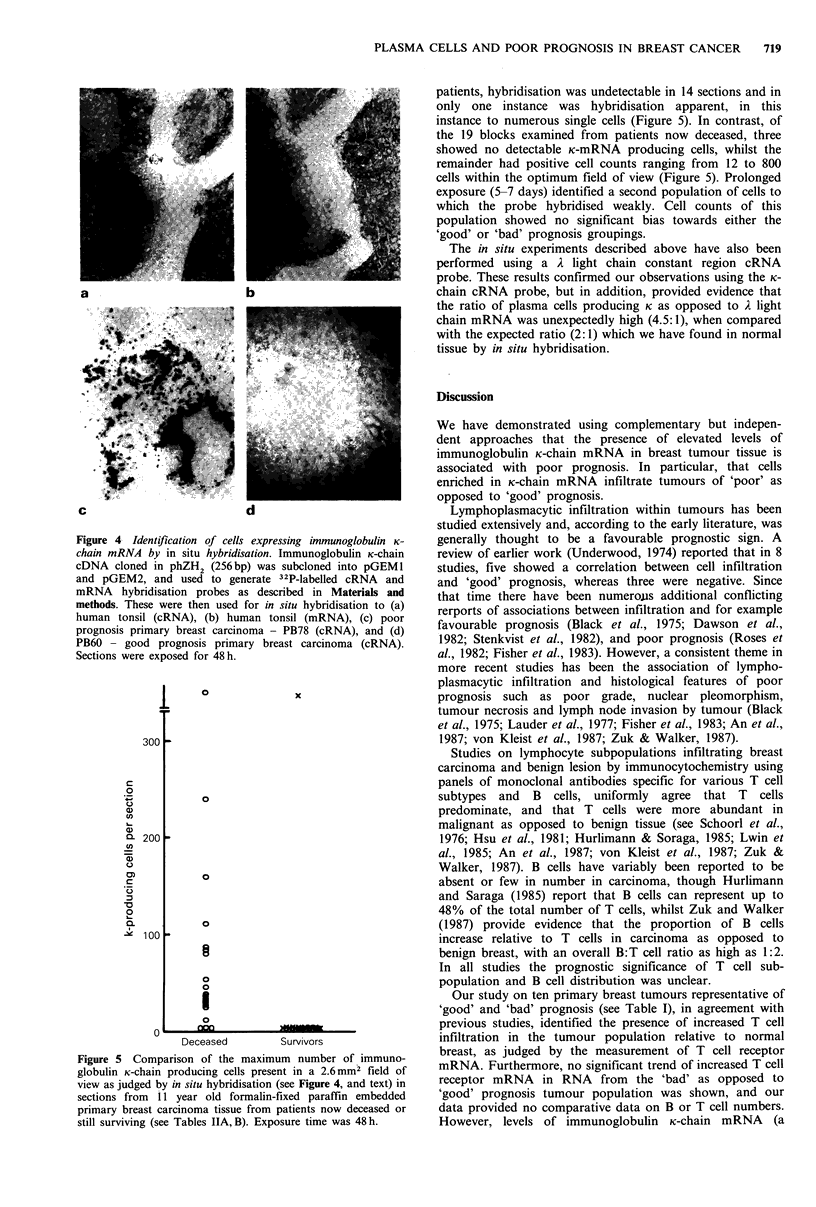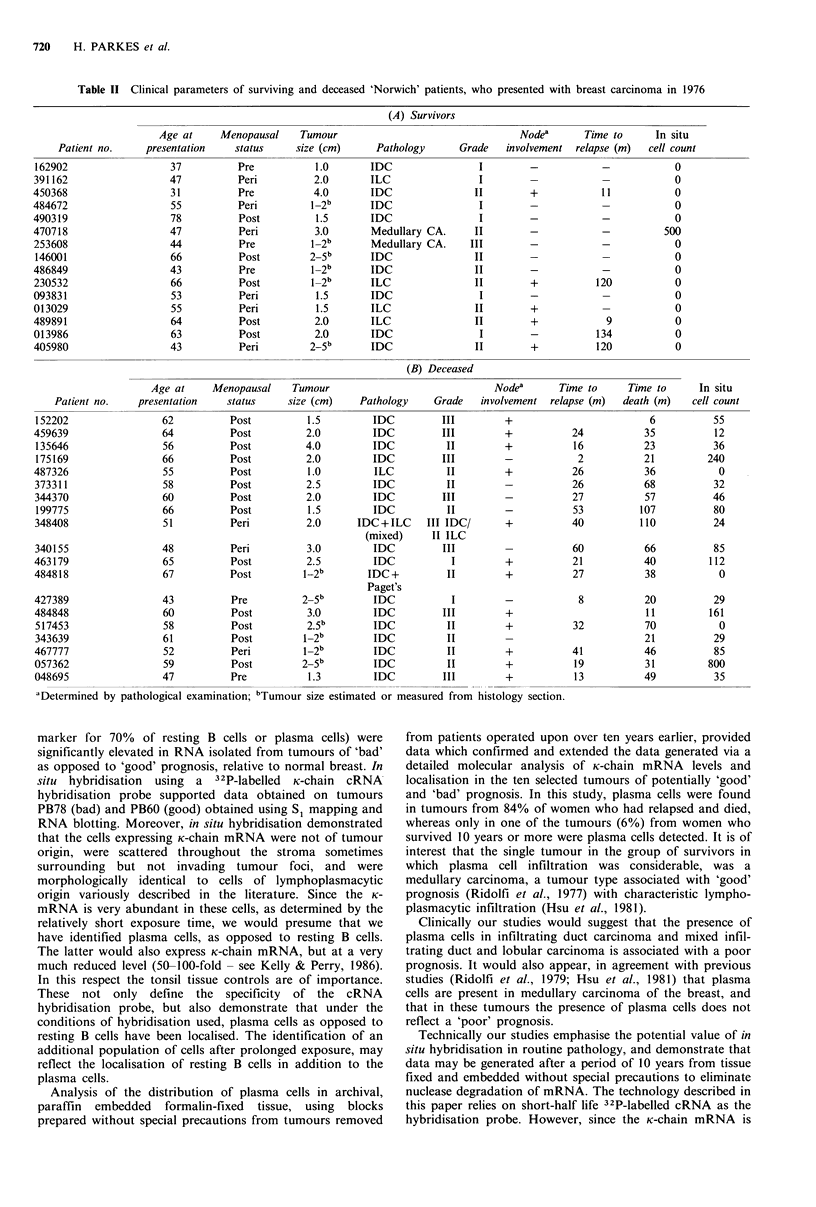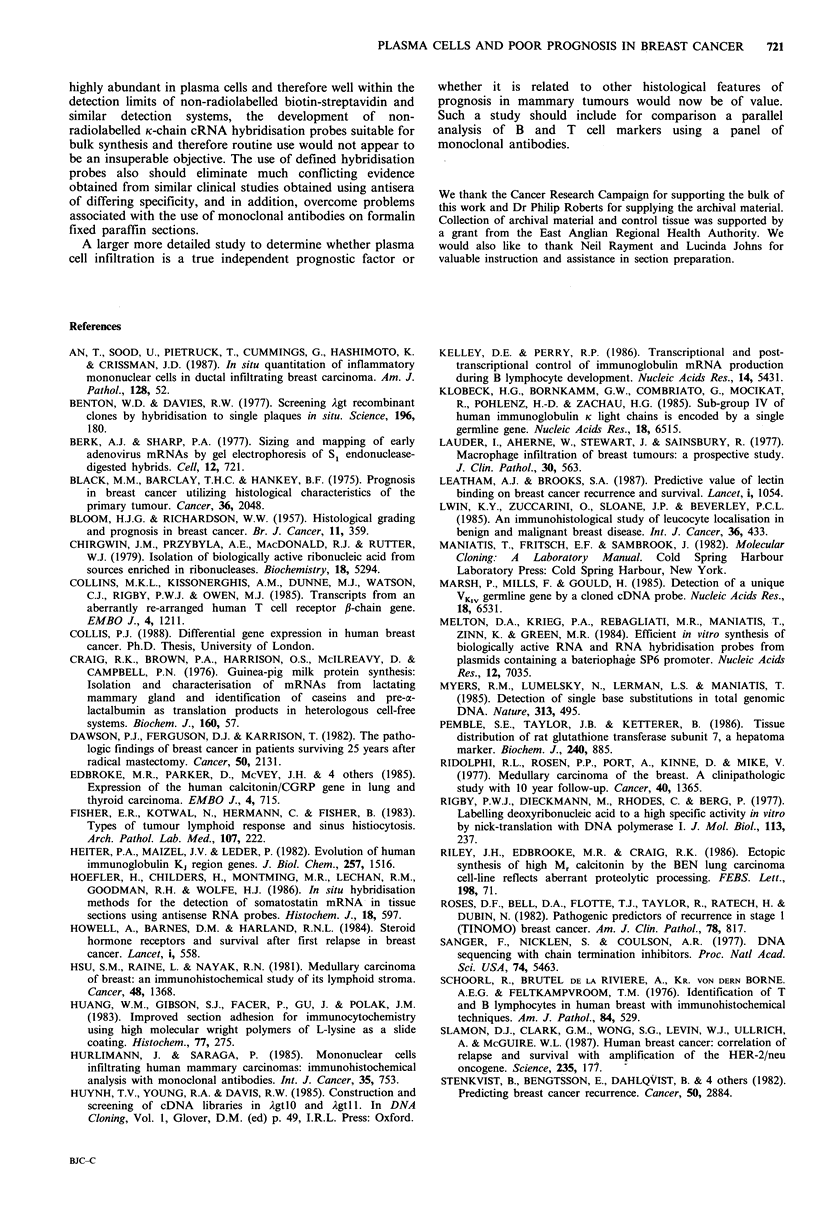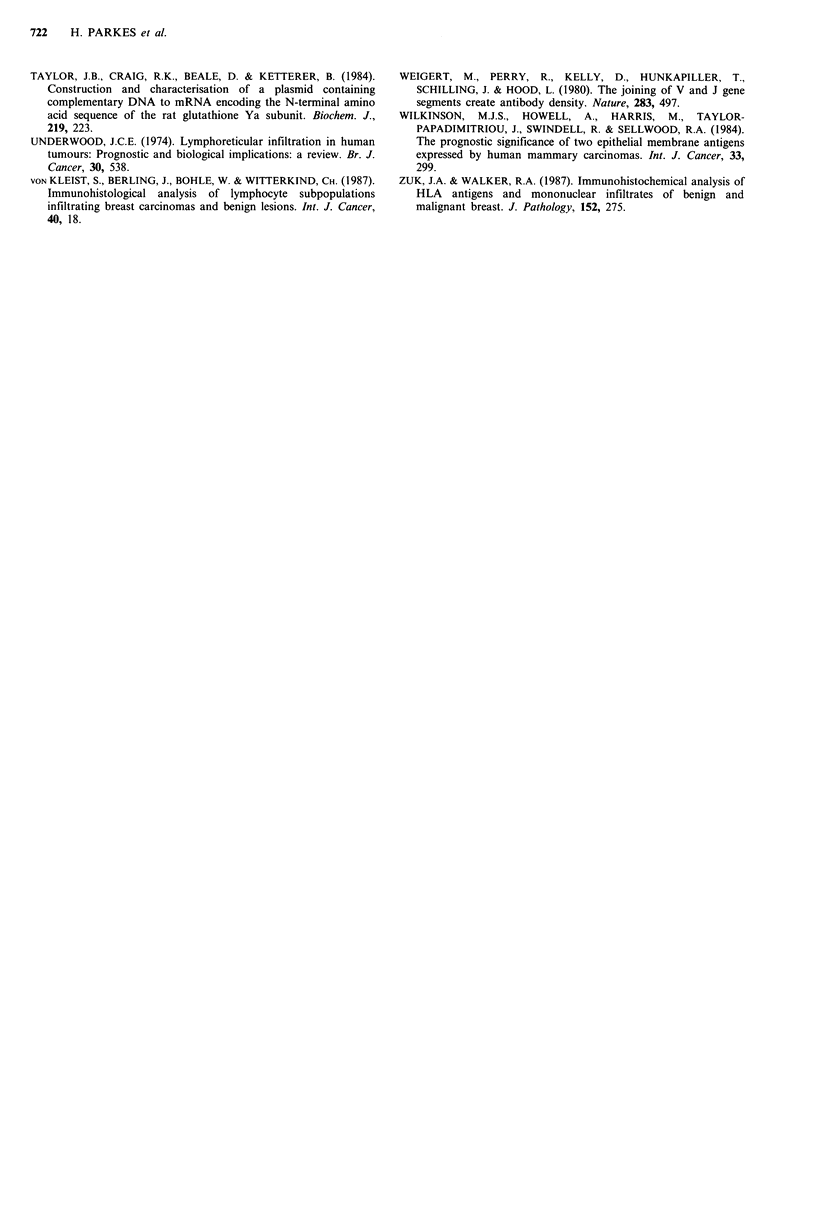# In situ hybridisation and S1 mapping show that the presence of infiltrating plasma cells is associated with poor prognosis in breast cancer.

**DOI:** 10.1038/bjc.1988.296

**Published:** 1988-12

**Authors:** H. Parkes, P. Collis, A. Baildam, D. Ralphs, B. Lyons, A. Howell, R. Craig

**Affiliations:** Cancer Research Campaign Tumour Endocrinology Group, Medical Molecular Biology Unit, University College and Middlesex School of Medicine, London, UK.

## Abstract

**Images:**


*Br. J. Cancer* (1988), **58**, 715–722
© The Macmillan Press Ltd., 1988

### *In situ* hybridisation and $S_1$ mapping show that the presence of infiltrating plasma cells is associated with poor prognosis in breast cancer

H. Parkes[1], P. Collis[1], A. Baildam[3], D. Ralphs[2] B. Lyons[2] A. Howell[3] & R. Craig[1]

[1]*The Cancer Research Campaign Tumour Endocrinology Group, The Medical Molecular Biology Unit, University College and Middlesex School of Medicine, London W1P 6DP;* [2]*Norfolk & Norwich Hospital, Norwich, Norfolk NR1 3SR; and* [3]*Cancer Research Campaign, Department of Medical Oncology, Christie Hospital and Holt Radium Institute, Manchester M20 9BX, UK.*

**Summary** In order to identify potential markers of prognosis in breast cancer, representative cDNA libraries were constructed using RNA isolated from primary breast tumour tissue associated with good and poor prognosis. Cross-screening of these libraries repeatedly identified cloned mRNA species associated with the immune system, in particular B-cells, in libraries derived from tumours of poor prognosis. We have used one of these, a $\kappa_{IV}$ light chain cDNA probe, in two complementary studies to investigate the relationship between immunoglobin gene expression and prognosis. The results obtained using a combination of $S_1$ mapping, RNA blotting and *in situ* hybridisation demonstrate that the presence of plasma cells, as defined by infiltrating cells which express high levels of immunoglobulin $\kappa$-chain mRNA, is associated with a poor prognosis.

A large proportion of women with operable primary breast cancer have occult micrometastases at presentation. The most accurate indicator of the presence of metastases is the extent of axillary lymph node involvement assessed by a pathologist after complete axillary dissection. However the introduction of breast conservation has reduced axillary dissections and the prognostic information derived from the determination of nodal involvement. This has led to a search for useful prognostic markers of metastases present within the primary tumour tissue such as histological grade (Richardson & Bloom, 1957), steroid receptor proteins (Howell *et al.*, 1984), milk fat globule antigens (Wilkinson *et al.*, 1984), *neu* oncogene expression (Slamon *et al.*, 1987) and cell surface glycoproteins (Leatham & Brooks, 1987). However, none of these markers is sufficiently accurate for clinical use, and new, more precise markers of prognosis would be of great value in order to plan appropriate treatments, and for the psychological management of the patient and her family.

In a series of experiments designed to identify novel markers of poor prognostic significance we constructed large cDNA libraries in bacteriophage λgt10 using poly(A)-containing RNA isolated from primary breast tumour tissue associated with good and poor prognosis. Cross-screening of these libraries repeatedly identified mRNA sequences associated with the immune system, in particular B-cells, in the cDNA library constructed from poor prognosis tumour tissue, suggesting a potential correlation between the presence of infiltrating cells of the immune system and poor prognosis. Here we describe $S_1$ mapping, RNA blotting and *in situ* hybridisation studies using a cloned human immunoglobulin light $\kappa$-chain cDNA hybridisation probe. These provide evidence that the presence of plasma cells, as defined by infiltrating cells producing high levels of immunoglobulin $\kappa$-chain mRNA, is associated with poor prognosis in primary mammary carcinoma.

### Materials and methods

### *Reagents*

[$\alpha$-$^{32}$P]dCTP (800 Ci mmol$^{-1}$) and [$\alpha$-$^{32}$P]CTP (800 Ci mmol$^{-1}$) were obtained from New England Nuclear (Boston, MA, USA). Restriction endonucleases and modifying enzymes were purchased from Boehringer Corporation Ltd. (Lewes, UK) or Amersham International plc (Amersham, UK). The cloning vectors pUC13, M13 mp10 and M13 mp11 were purchased from Pharmacia (Uppsala, Sweden) whilst pGEM 1 and pGEM 2 were from P & S Biochemicals (Liverpool, UK). All other materials were from sources described elsewhere (Edbrooke *et al.*, 1985; Pemble *et al.*, 1986).

### *Patients and pathological material*

Primary breast tumour tissue for RNA extraction was obtained from patients involved in clinical trials at The Christie Hospital and Holt Radium Institute, Manchester. For retrospective studies, formalin fixed, paraffin embedded breast carcinoma tissue was obtained from the Department of Pathology, Norfolk & Norwich General Hospital, Norwich. Tissue for RNA extraction was frozen in liquid nitrogen immediately after excision. For *in situ* hybridisation studies formalin fixed tissues were processed and embedded in paraffin wax in routine pathology laboratories, without special precautions to prevent RNA degradation, then stored at ambient temperature for up to 11 years.

### *RNA isolation*

Total RNA was extracted from frozen tumour tissue essentially as described by Chirgwin *et al.* (1979). Briefly, up to 1 g of frozen tissue was ground to powder under liquid $N_2$ and then homogenized in 10 ml of guanidine isothiocyanate buffer (50 mM Tris/HCl pH 7.6, containing 10 mM EDTA, 2% (w/v) sodium lauryl sarcosine, 0.01% (v/v) β mercapto-ethanol, and 4 M guanidine isothiocyanate). Debris was cleared from the homogenate by centrifugation at 10,000 *g* for 10 min at 10°C, and the supernatant overlayed onto a 3 ml CsCl cushion (5.7 M CsCl, 100 mM EDTA pH 8.0). This was centrifuged in a Beckman SW40 rotor at 30,000 rpm for 18 h at 20°C. Following centrifugation, the supernatant was discarded and the RNA pellet rinsed with 70% (v/v) ethanol and resuspended in 1 ml of guanidine HCl buffer (7 M guanidine HCl, 10 mM sodium iodoacetate, 20 mM sodium acetate, 1 mM dithiothreitol, 20 mM EDTA pH 7.0). RNA was precipitated directly from this buffer by addition of half a volume of absolute ethanol, at −20°C overnight. RNA was recovered by centrifugation, and reprecipitated twice before being resuspended in 10 mM Tris HCl pH 8.0, 1 mM EDTA and stored at −70°C.

The poly(A)-containing RNA was isolated from total

Correspondence: R.K. Craig.
Received 1 March 1988; and in revised form 9 June 1988.

cellular RNA by affinity chromatography on oligo-dT cellulose, essentially as described by Craig et al. (1976), except that column equilibration and binding was performed in 10 mM HEPES pH 7.5 containing 500 mM lithium chloride, and 0.2% (w/v) SDS, while elution was with 10 mM HEPES pH 7.5 containing 0.2% (w/v) SDS at 60°C.

*Construction and screening of mammary carcinoma cDNA libraries in λgt10*

Representative cDNA libraries were prepared from total poly(A)-containing RNA isolated from breast carcinoma tissue. Double stranded cDNA was synthesised from 2 μg poly(A)-containing RNA, according to conditions described by Riley et al. (1986). Subsequent modification of the cDNA, ligation to EcoRI restricted λgt10 DNA and packaging into λgt10 phage particles was as detailed by Huynh et al. (1985).

Cross-screening of the cDNA libraries using $^{32}P$-labelled cDNA was carried out at low density ($1-2 \times 10^3$ pfu/9 cm plate) using the hybridisation conditions described by Benton & Davis (1977). Individual differentially expressed recombinant phage plaques were picked, amplified in liquid culture, and cDNA inserts excised and subcloned directly into the EcoRI site of pUC 13 using procedures described by Maniatis et al. (1982). EcoRI excised inserts from this subclone were then ligated into M13 vectors (Mp10, Mp11) for DNA sequence analysis (Sanger et al., 1977) and $S_1$ mapping experiments. Inserts were also recloned into Gemini vectors (pGEM 1, pGEM 2) for preparation of RNA transcripts for *in situ* hybridisation.

*RNA blotting and $S_1$ mapping*

Analysis of poly(A)-containing RNA by RNA blotting was carried out as described by Taylor et al. (1984). Probes were labelled with [$\alpha^{32}P$]dCTP by nick translation (Rigby et al., 1977).

$S_1$ mapping experiments were performed employing conditions modified from Berk & Sharp (1977), using a single stranded $^{32}P$-labelled M13 DNA probe, prepared essentially as described by Myers et al. (1985). Hybridisation reactions were incubated at 52°C for 18 h, and $S_1$ nuclease digestion was at 37°C for 1 h. Protected fragments were analysed by electrophoresis on 6% (w/v) polyacrylamide urea gels.

In situ *hybridisation*

Tumour blocks were sectioned (5μ) on a microtome and sections layered onto clean, sterile, poly-L-lysine coated glass slides (Huang et al., 1983). These were baked at 37°C overnight prior to storage at room temperature in a dry, dust-free box. Sections stored in this manner have been successfully used over a period of 6 months. Prior to use sections were dewaxed by sequential immersion of slides in xylene, followed by stepwise rehydration through alcohol. Further pre-treatment of sections prior to *in situ* hybridisation, was performed as detailed by Hoefler et al. (1986).

Radiolabelled sense and anti-sense RNA probes were synthesised from HindIII linearised pGEM 1 (cRNA) and pGEM 2 (mRNA) constructs, by incorporation of [$\alpha$-$^{32}P$]CTP into transcripts from the T7 promoter, as modified by Promega (technical bulletin) from Melton et al. (1984). RNA probe (4 ng), corresponding to $2 \times 10^6$ cpm, was applied to each section in 20 μl hybridisation buffer. Hybridisation was performed as described by Hoefler et al. (1986). After washing and dehydration, the slides were coated in Ilford liquid nuclear track emulsion (K5), dried and exposed at 4°C in light-tight boxes for 2–10 days. Slides were developed at 20°C in Kodak D19 developer, and fixed in 5% (w/v) sodium thiosulphate. Sections were counterstained with haematoxylin and eosin prior to microscopic examination.

### Results

*Identification of κ-mRNA in RNA preparations from primary breast carcinoma*

We have constructed a number of representative λgt10 cDNA libraries ($2-3 \times 10^6$ recombinants) from poly(A)-containing RNA isolated from primary breast carcinomas containing oestrogen and progesterone receptors or no receptors, and from primary breast carcinomas which on the criteria of size, grade, lymph node involvement, extracellular HMFG staining, and receptor status, are indicative of good or bad prognosis (see Table I). Direct cross-screening of these libraries with $^{32}P$-labelled cDNA probes using strategies to identify cloned mRNA species prevalent for example in the tumour of potentially bad as opposed to good prognosis, repeatedly resulted in the identification of immunoglobulin κ-light and γ-heavy chain mRNA sequences, data consistent with the view that there was significant infiltration by B-cells of the 'poor' prognosis tumour relative to that of 'good' prognosis (Collis, 1988).

In order to investigate the possibility that levels of immunoglobulin mRNA expression might prove a useful prognostic factor, we have used one of these cloned sequences (phZH2) as a hybridisation probe to quantitate and localise the site of immunoglobulin mRNA expression in breast cancer. The cloned cDNA inserted in phZH2 (Figure 1) comprises a 256 bp fragment of κ light chain mRNA, containing an open reading frame, encoding part of the variable region ($V_k$), J-region ($J_k$) and part of the constant region ($C_k$). Comparison of the deduced amino acid sequence with other available K-chain amino sequences in the $V_k$ regions demonstrated amino acid sequence homology with κ subgroup IV (see Klobeck et al., 1985; Marsh et al., 1985). We have used this $\kappa_{IV}$ cDNA sequence, recloned into

**Table I** Clinical parameters used to define tumours of potentially good and bad prognosis

| | *Poor*[a] | | | | | *Good*[a] | | | | |
|---|---|---|---|---|---|---|---|---|---|---|
| Patient no. | 13 | 19 | 44 | 76 | 78 | 60 | 92 | 127 | 130 | 132 |
| Age | 54 | 53 | 67 | 54 | 73 | 64 | 49 | 58 | 37 | 76 |
| Menopausal status | Pre | Pre | Post | Pre | Post | Post | Pre | Post | Pre | Post |
| Tumour size (cm) | 10 | 6 | 6 | 8 | 2 | 6 | 3 | 3 | 2 | 1.5 |
| Node involvement | + | − | + | + | − | − | − | − | − | − |
| Pathology[b] | IDC | IDC | IDC | IDC | IDC | IDC | IDC | IDC/ILC | IDC | IDC/ILC |
| Grade[c] | III | III | III | III | III | I | I | ND | I | ND |
| Receptors ER/PR | −/− | +/− | +/+ | −/− | −/− | +/+ | +/+ | +/− | −/+ | +/+ |
| Extracellular milk fat globule antigen (ECS)[d] | − | − | − | − | − | + | + | + | + | − |
| Rime to relapse (m) | 12 | 10 | 7 | 1 | 7 | − | − | 3 | − | − |
| Time to death (m) | 21 | 32 | − | 14 | 14 | − | − | − | − | − |

[a]Selected on basis of tumour size, grade, nodal involvement, ECS with HMFG and disease free interval; [b]Infiltrating duct carcinoma (IDC). Mixed infiltrating duct/infiltrating lobular carcinoma IDC/ILC; [c]Richardson & Bloom (1957); [d]Wilkinson et al. (1984). We took ECS to be a good prognostic feature.

```
                                        Vk
5' THR LEU THR ILE SER SER LEU GLN ALA GLU ASP ALA ALA VAL TYR TYR CYS GLN GLN TYR
   ACT CTC ACC ATC AGC AGC CTG CAG GCT GAA GAT GCG GCA GTT TAT TAC TGT CAG CAA TAT    60
                                        Jk
   VAL SER THR PRO|ARG ALA PHE GLY PRO GLY THR LYS VAL GLU ILE LYS ARG|THR VAL ALA
   GTG AGT ACT CCT|CGT GCA TTC GGC CCA GGG ACC AAG GTG GAA ATC AAA CGA|ACT GTG GCT   120

   ALA PRO SER VAL PHE ILE PHE PRO PRO SER ASP GLU GLN LEU LYS SER GLY THR ALA SER
   GCA CCA TCT GTC TTC ATC TTC CCG CCA TCT GAT GAG CAG TTG AAA TCT GGA ACT GCC TCT   180
                                        Ck
   VAL VAL CYS LEU LEU ASN ASN PHE TYR PRO ARG GLU ALA LYS VAL GLN TRP LYS VAL ASP
   GTT GTG TGC CTG CTG AAT AAC TTC TAT CCC AGA GAG GCC AAA GTA CAG TGG AAG GTG GAT   240

   ASN ALA LEU GLN SER
   AAC GCC CTC CAA TCG 3'                                                             255
```

**Figure 1** *Nucleotide sequence of immunoglobulin* $\kappa_{IV}$ *light chain mRNA cloned in phZH2.* Nucleotide sequence analysis was performed on both stands and across all restriction sites used in the sequencing strategy as described in **Materials and methods**. Deduced amino acid sequence encoding variable region ($V_K$), J-region ($V_J$), and constant region ($C_K$) is indicated.

appropriate vectors, to investigate by RNA blotting, $S_1$ mapping and *in situ* hybridisation the relative levels, and site of synthesis of $\kappa$-chain mRNA in RNA isolated from primary breast carcinomas, which, on a number of criteria (see Table I) were predicted to be of 'good' or 'bad' prognosis. This selection was found later to be reasonably accurate since on follow up all the tumours with features of a poor prognosis have resulted in relapse in all patients and death in 4 out of 5 within 32 months, whereas those with features of a good prognosis are alive 30–40 months after mastectomy, of which only one has relapsed (see Table I).

Initially the levels of $\kappa$-chain mRNA expressed in tumour RNA were assessed by RNA (Northern) blotting relative to actin mRNA in a small group of tumours. Total poly(A)-containing RNA was separated on the basis of size by gel electrophoresis, blotted onto Biodyne membranes and co-probed with $^{32}$P-labelled phZH2 and pAM-91 (actin) cDNA probes. A typical result (Figure 2), demonstrates the presence of an intense band of the expected size of $\kappa$-chain mRNA (1100 ntds) in three RNA preparations all from tumours of bad prognosis, the presence of the same band in the control tonsil RNA preparation, and the presence of low levels only of $\kappa$-chain mRNA in the two good prognosis tumours examined in this instance. The actin mRNA (1600 ntds) was present at approximately equivalent levels in all RNA preparations.

In order to increase the sensitivity of our analyses, conserve RNA, and to determine the relative level of expression of $K_{IV}$ mRNA to total $\kappa$-mRNA, we have performed $S_1$ mapping using a 327 ntd uniformly $^{32}$P-radiolabelled M13 derived cDNA probe containing M13

**Figure 2** *Identification of immunoglobulin* $\kappa$*-chain mRNA in RNA isolated from primary human breast carcinoma by RNA blotting.* Total poly (A) containing RNA (1 $\mu$g per track) isolated from (i) human tonsil (T), and (ii–vi) five human primary breast carcinoma of good (PB60, PB92) and bad (PB44, PB19, PB78 – see **Table I**) prognosis, was analysed by RNA blotting for the presence of immunoglobulin $\kappa$-light chain mRNA and actin mRNA (see text and **Materials and methods**). The position of DNA size markers of 1631, 517, 396 bp, and $\kappa$-light chain and actin mRNA is indicated.

polylinker sequence, and sequence encoding $V_{kIV}$, $J_k$ and $C_k$ (see Figure 3AI). The results (Figure 3BI) compare $\kappa$-mRNA levels in poly(A)-containing RNA isolated from five bad prognosis, and five good prognosis tumours (see Table I) with RNA from a tonsil (positive control), benign breast and normal breast. In the tonsil control track, a number of diffuse protected bands are apparent, the two most prominent being of 183 and 175 ntds, representing complete protection of the $C_k$ and $J_k$ region of the probe, and limited $S_1$ nuclease digestion of part of the 5′ end of the $J_k$ region respectively. This was expected taking into account J region duplication (Hieter *et al.*, 1982) and junctional variation during V–J recombination (Weigert *et al.*, 1980). Some larger but minor species were also present. These represent $K_{IV}$ mRNA (256 ntds) and protection of limited sequence in the $V_k$ region in addition to $J_k$ and $C_k$ sequence (194 ntds). Comparative analysis of all breast RNA preparations, demonstrated that relative $\kappa$-mRNA levels in the five 'bad' prognosis tumours were 5–30-fold higher than $\kappa$-mRNA levels in the normal breast RNA preparations. In a comparison of the 'good' prognosis tumours, three showed barely detectable levels of $\kappa$-mRNA, and two had $\kappa$-mRNA levels 2–4-fold higher than normal breast RNA. $\kappa_{IV}$ mRNA was identified at low levels, in tonsil and two 'bad prognosis tumours (PB13, PB44). The absence of detectable $\kappa_{IV}$ mRNA in the remaining RNA preparations probably reflects the low levels of total $\kappa$-mRNA of which $\kappa_{IV}$ mRNA is a minor component.

In addition to our analysis of the relative levels of $\kappa$-mRNA, a potential marker for plasma cells in the tumour tissues, we have also examined by $S_1$ mapping in the same RNA preparations (see Figure 3AII), the relative levels of T-cell receptor $\beta$-chain mRNA (Collins *et al.*, 1985), an equivalent marker for T cells. The results (Figure 3BII), using the same RNA samples used for $\kappa$-mRNA analysis, show the expected protected T cell receptor mRNA band of 479 ntds in the tonsil RNA preparation, identifying the presence of T cell receptor mRNA. The protected band was barely discernible in the benign and normal breast RNA preparations, and of the remainder, only in one 'good' prognosis RNA preparation were T cell receptor mRNA levels significantly increased (2–3-fold) compared with normal breast. Analysis of T cell receptor mRNA levels in RNA preparations from bad prognosis tumours, showed a small 2–3-fold increase, and in one instance a 10–15-fold increase in T cell receptor mRNA levels relative to normal breast. Overall T cell receptor mRNA levels were elevated in tumour tissue, but there was no particular bias between good and bad prognosis tumour RNA preparations.

### *Retrospective analysis of* $\kappa$*-mRNA expression by* in situ *hybridisation*

The results described above on a relatively small number of clinically well defined breast tumour preparations, provide evidence for increased infiltration of primary breast tumours, where the prognosis is poor, by cells of the immune system, in particular B cells. The approach whilst revealing, is time consuming, too complex for the routine pathology laboratory, and inappropriate for retrospective studies where available material is limited to formalin fixed paraffin embedded blocks. We have therefore employed *in situ* hybridisation using $^{32}$P-labelled $\kappa$-chain cRNA hybridisation probes to identify $\kappa$-mRNA producing cells in paraffin embedded formalin fixed tissue sections. Initially the validity of the technique was established using tonsil, rapidly processed post-operatively. As can be seen the morphology of the section is retained in spite of prolonged hybridisations (Figure 4a), and the $\kappa$-chain cRNA probe hybridises strongly to discrete regions of the tonsil tissue (as judged by autoradiography) consistent with the presence of a cuff of mature plasma cells around reactive follicles. In contrast the $^{32}$P-labelled $\kappa$-chain mRNA probe showed no hybridisation to

**Figure 3** *$S_1$ analysis of the relative amounts of immunoglobulin κ-light chain mRNA and T cell receptor mRNA in RNA isolated from human primary breast carcinoma.* (A) Single stranded $^{32}$P-labelled cDNA probes specific for immunoglobulin κ-chain mRNA species (I) and TiCβ chain mRNA of the T cell receptor (II) were generated by recloning phZH$_2$ cDNA (256 bp) and 479 bp EcoRI fragment of PB400 into M13 (see Collins *et al*., 1985, and **Materials and methods**), resulting in cDNA hybridisation probes of 327 and 550 ntds respectively. (B) $S_1$ nuclease analysis of poly (A) containing RNA (500 ng per track) using (I) a $\kappa_{IV}$ cDNA probe, or (II) a T cell receptor β-chain cDNA probe was carried out as described in **Materials and methods**. Samples were analysed in the following order; track (i) tonsil RNA, tracks (ii–vi) poor prognosis tumour RNA (PB76, 13, 78, 19, and 44 respectively), tracks (vii–xi) good prognosis tumour RNA (PB 132, 130, 127, 92 and 60 respectively) track (xii) benign breast carcinoma RNA, and track (xiii) normal breast RNA. The relative mobility of Hinf I restricted pAT153 size markers of 298, 220 and 154 pb, and the size of bands resistant to $S_1$ nuclear digestion are shown.

tonsil tissue, thereby demonstrating the specificity of the hybridisation probe (Figure 4b). Application of the same approach to formalin fixed paraffin embedded sections representative of good (PB60) and bad (PB78) prognosis primary breast tissues, in this instance using sections cut from blocks prepared without special precautions in a routine hospital pathology environment, showed results consistent with data obtained by RNA blotting and $S_1$ mapping of RNA isolated from the same tissues (see Figures 2 and 3). Hybridisation of the $^{32}$P-labelled κ-chain cRNA probe to the bad prognosis tissue was intense and localised to single cells scattered throughout the section, but focussed in the stroma immediately surrounding although generally not invading the tumour foci (Figure 4c). No hybridisation was observed to any cells on examination of sections of good prognosis tissue (PB60) which were processed in parallel to the bad prognosis (PB78) using the same cRNA probe preparations, hybridisation and autoradiographic solutions (Figure 4d). In all experiments the control $^{32}$P-labelled κ-mRNA hybridisation probe showed no hybridisation to any section when analysed in parallel and developed after an identical period of exposure. In some instances regions of 'yellowing' were seen over single cells. Examination under dark field microscopy showed no evidence for silver grains in these regions, which we presume therefore may reflect an artefact of the methodology. On the basis of the data presented it would appear that *in situ* hybridisation can be successfully applied to formalin fixed paraffin embedded tissue sections, producing results in agreement with parallel analysis of κ-chain RNA expression by RNA blotting and $S_1$ mapping in RNA isolated from snap frozen tissue from the same tumours.

Having validated the technology and established the potential prognostic value of the presence of plasma cells in tumour tissue using a limited number of primary tumours obtained from a relatively recent study, we have extended our analysis to examine by *in situ* hybridisation the presence of κ-mRNA producing cells in paraffin sections taken from 34 formalin fixed primary breast carcinomas removed in 1976 at the Norfolk and Norwich Hospital. The tumours were selected on the criteria that approximately half ($n = 19$) were from patients who had succumbed to the disease during a 10 year post-operative period, and the remainder were from patients who were alive, and in some instances had no recurrent disease, during the same period ($n = 15$) – see Table IIA, B. Sections were cut from all the blocks, and the number of κ-mRNA producing cells in each grouping examined by *in situ* hybridisation. Sections were scanned under light microscopy, and the area of tissue within a 2.6 mm$^2$ field of view containing the greatest number of κ-mRNA producing cells scored blind. After a 48 h exposure period, out of the 15 blocks examined from the surviving

**Figure 4** *Identification of cells expressing immunoglobulin κ-chain mRNA by* in situ *hybridisation.* Immunoglobulin κ-chain cDNA cloned in $phZH_2$ (256 bp) was subcloned into pGEM1 and pGEM2, and used to generate $^{32}P$-labelled cRNA and mRNA hybridisation probes as described in **Materials and methods**. These were then used for *in situ* hybridisation to (a) human tonsil (cRNA), (b) human tonsil (mRNA), (c) poor prognosis primary breast carcinoma – PB78 (cRNA), and (d) PB60 – good prognosis primary breast carcinoma (cRNA). Sections were exposed for 48 h.

**Figure 5** Comparison of the maximum number of immunoglobulin κ-chain producing cells present in a 2.6 $mm^2$ field of view as judged by *in situ* hybridisation (see **Figure 4**, and text) in sections from 11 year old formalin-fixed paraffin embedded primary breast carcinoma tissue from patients now deceased or still surviving (see Tables IIA, B). Exposure time was 48 h.

patients, hybridisation was undetectable in 14 sections and in only one instance was hybridisation apparent, in this instance to numerous single cells (Figure 5). In contrast, of the 19 blocks examined from patients now deceased, three showed no detectable κ-mRNA producing cells, whilst the remainder had positive cell counts ranging from 12 to 800 cells within the optimum field of view (Figure 5). Prolonged exposure (5–7 days) identified a second population of cells to which the probe hybridised weakly. Cell counts of this population showed no significant bias towards either the 'good' or 'bad' prognosis groupings.

The *in situ* experiments described above have also been performed using a λ light chain constant region cRNA probe. These results confirmed our observations using the κ-chain cRNA probe, but in addition, provided evidence that the ratio of plasma cells producing κ as opposed to λ light chain mRNA was unexpectedly high (4.5:1), when compared with the expected ratio (2:1) which we have found in normal tissue by *in situ* hybridisation.

### Discussion

We have demonstrated using complementary but independent approaches that the presence of elevated levels of immunoglobulin κ-chain mRNA in breast tumour tissue is associated with poor prognosis. In particular, that cells enriched in κ-chain mRNA infiltrate tumours of 'poor' as opposed to 'good' prognosis.

Lymphoplasmacytic infiltration within tumours has been studied extensively and, according to the early literature, was generally thought to be a favourable prognostic sign. A review of earlier work (Underwood, 1974) reported that in 8 studies, five showed a correlation between cell infiltration and 'good' prognosis, whereas three were negative. Since that time there have been numerous additional conflicting rerports of associations between infiltration and for example favourable prognosis (Black *et al.*, 1975; Dawson *et al.*, 1982; Stenkvist *et al.*, 1982), and poor prognosis (Roses *et al.*, 1982; Fisher *et al.*, 1983). However, a consistent theme in more recent studies has been the association of lymphoplasmacytic infiltration and histological features of poor prognosis such as poor grade, nuclear pleomorphism, tumour necrosis and lymph node invasion by tumour (Black *et al.*, 1975; Lauder *et al.*, 1977; Fisher *et al.*, 1983; An *et al.*, 1987; von Kleist *et al.*, 1987; Zuk & Walker, 1987).

Studies on lymphocyte subpopulations infiltrating breast carcinoma and benign lesion by immunocytochemistry using panels of monoclonal antibodies specific for various T cell subtypes and B cells, uniformly agree that T cells predominate, and that T cells were more abundant in malignant as opposed to benign tissue (see Schoorl *et al.*, 1976; Hsu *et al.*, 1981; Hurlimann & Soraga, 1985; Lwin *et al.*, 1985; An *et al.*, 1987; von Kleist *et al.*, 1987; Zuk & Walker, 1987). B cells have variably been reported to be absent or few in number in carcinoma, though Hurlimann and Saraga (1985) report that B cells can represent up to 48% of the total number of T cells, whilst Zuk and Walker (1987) provide evidence that the proportion of B cells increase relative to T cells in carcinoma as opposed to benign breast, with an overall B:T cell ratio as high as 1:2. In all studies the prognostic significance of T cell subpopulation and B cell distribution was unclear.

Our study on ten primary breast tumours representative of 'good' and 'bad' prognosis (see Table I), in agreement with previous studies, identified the presence of increased T cell infiltration in the tumour population relative to normal breast, as judged by the measurement of T cell receptor mRNA. Furthermore, no significant trend of increased T cell receptor mRNA in RNA from the 'bad' as opposed to 'good' prognosis tumour population was shown, and our data provided no comparative data on B or T cell numbers. However, levels of immunoglobulin κ-chain mRNA (a

**Table II** Clinical parameters of surviving and deceased 'Norwich' patients, who presented with breast carcinoma in 1976

| | (*A*) *Survivors* | | | | | | | |
|---|---|---|---|---|---|---|---|---|
| *Patient no.* | *Age at presentation* | *Menopausal status* | *Tumour size (cm)* | *Pathology* | *Grade* | *Node*[a] *involvement* | *Time to relapse (m)* | In situ *cell count* |
| 162902 | 37 | Pre | 1.0 | IDC | I | – | – | 0 |
| 391162 | 47 | Peri | 2.0 | ILC | I | – | – | 0 |
| 450368 | 31 | Pre | 4.0 | IDC | II | + | 11 | 0 |
| 484672 | 55 | Peri | 1–2[b] | IDC | I | – | – | 0 |
| 490319 | 78 | Post | 1.5 | IDC | I | – | – | 0 |
| 470718 | 47 | Peri | 3.0 | Medullary CA. | II | – | – | 500 |
| 253608 | 44 | Pre | 1–2[b] | Medullary CA. | III | – | – | 0 |
| 146001 | 66 | Post | 2–5[b] | IDC | II | – | – | 0 |
| 486849 | 43 | Pre | 1–2[b] | IDC | II | – | – | 0 |
| 230532 | 66 | Post | 1–2[b] | ILC | II | + | 120 | 0 |
| 093831 | 53 | Peri | 1.5 | IDC | I | – | – | 0 |
| 013029 | 55 | Peri | 1.5 | ILC | II | + | – | 0 |
| 489891 | 64 | Post | 2.0 | ILC | II | + | 9 | 0 |
| 013986 | 63 | Post | 2.0 | IDC | I | – | 134 | 0 |
| 405980 | 43 | Peri | 2–5[b] | IDC | II | + | 120 | 0 |

| | (*B*) *Deceased* | | | | | | | | |
|---|---|---|---|---|---|---|---|---|---|
| *Patient no.* | *Age at presentation* | *Menopausal status* | *Tumour size (cm)* | *Pathology* | *Grade* | *Node*[a] *involvement* | *Time to relapse (m)* | *Time to death (m)* | In situ *cell count* |
| 152202 | 62 | Post | 1.5 | IDC | III | + | | 6 | 55 |
| 459639 | 64 | Post | 2.0 | IDC | III | + | 24 | 35 | 12 |
| 135646 | 56 | Post | 4.0 | IDC | II | + | 16 | 23 | 36 |
| 175169 | 66 | Post | 2.0 | IDC | III | – | 2 | 21 | 240 |
| 487326 | 55 | Post | 1.0 | ILC | II | + | 26 | 36 | 0 |
| 373311 | 58 | Post | 2.5 | IDC | II | – | 26 | 68 | 32 |
| 344370 | 60 | Post | 2.0 | IDC | III | – | 27 | 57 | 46 |
| 199775 | 66 | Post | 1.5 | IDC | II | – | 53 | 107 | 80 |
| 348408 | 51 | Peri | 2.0 | IDC+ILC (mixed) | III IDC/ II ILC | + | 40 | 110 | 24 |
| 340155 | 48 | Peri | 3.0 | IDC | III | – | 60 | 66 | 85 |
| 463179 | 65 | Post | 2.5 | IDC | I | + | 21 | 40 | 112 |
| 484818 | 67 | Post | 1–2[b] | IDC+ Paget's | II | + | 27 | 38 | 0 |
| 427389 | 43 | Pre | 2–5[b] | IDC | I | – | 8 | 20 | 29 |
| 484848 | 60 | Post | 3.0 | IDC | III | + | | 11 | 161 |
| 517453 | 58 | Post | 2.5[b] | IDC | II | + | 32 | 70 | 0 |
| 343639 | 61 | Post | 1–2[b] | IDC | II | – | | 21 | 29 |
| 467777 | 52 | Peri | 1–2[b] | IDC | II | + | 41 | 46 | 85 |
| 057362 | 59 | Post | 2–5[b] | IDC | II | + | 19 | 31 | 800 |
| 048695 | 47 | Pre | 1.3 | IDC | III | + | 13 | 49 | 35 |

[a]Determined by pathological examination; [b]Tumour size estimated or measured from histology section.

marker for 70% of resting B cells or plasma cells) were significantly elevated in RNA isolated from tumours of 'bad' as opposed to 'good' prognosis, relative to normal breast. *In situ* hybridisation using a $^{32}$P-labelled $\kappa$-chain cRNA hybridisation probe supported data obtained on tumours PB78 (bad) and PB60 (good) obtained using $S_1$ mapping and RNA blotting. Moreover, *in situ* hybridisation demonstrated that the cells expressing $\kappa$-chain mRNA were not of tumour origin, were scattered throughout the stroma sometimes surrounding but not invading tumour foci, and were morphologically identical to cells of lymphoplasmacytic origin variously described in the literature. Since the $\kappa$-mRNA is very abundant in these cells, as determined by the relatively short exposure time, we would presume that we have identified plasma cells, as opposed to resting B cells. The latter would also express $\kappa$-chain mRNA, but at a very much reduced level (50–100-fold – see Kelly & Perry, 1986). In this respect the tonsil tissue controls are of importance. These not only define the specificity of the cRNA hybridisation probe, but also demonstrate that under the conditions of hybridisation used, plasma cells as opposed to resting B cells have been localised. The identification of an additional population of cells after prolonged exposure, may reflect the localisation of resting B cells in addition to the plasma cells.

Analysis of the distribution of plasma cells in archival, paraffin embedded formalin-fixed tissue, using blocks prepared without special precautions from tumours removed from patients operated upon over ten years earlier, provided data which confirmed and extended the data generated via a detailed molecular analysis of $\kappa$-chain mRNA levels and localisation in the ten selected tumours of potentially 'good' and 'bad' prognosis. In this study, plasma cells were found in tumours from 84% of women who had relapsed and died, whereas only in one of the tumours (6%) from women who survived 10 years or more were plasma cells detected. It is of interest that the single tumour in the group of survivors in which plasma cell infiltration was considerable, was a medullary carcinoma, a tumour type associated with 'good' prognosis (Ridolfi *et al.*, 1977) with characteristic lymphoplasmacytic infiltration (Hsu *et al.*, 1981).

Clinically our studies would suggest that the presence of plasma cells in infiltrating duct carcinoma and mixed infiltrating duct and lobular carcinoma is associated with a poor prognosis. It would also appear, in agreement with previous studies (Ridolfi *et al.*, 1979; Hsu *et al.*, 1981) that plasma cells are present in medullary carcinoma of the breast, and that in these tumours the presence of plasma cells does not reflect a 'poor' prognosis.

Technically our studies emphasise the potential value of *in situ* hybridisation in routine pathology, and demonstrate that data may be generated after a period of 10 years from tissue fixed and embedded without special precautions to eliminate nuclease degradation of mRNA. The technology described in this paper relies on short-half life $^{32}$P-labelled cRNA as the hybridisation probe. However, since the $\kappa$-chain mRNA is highly abundant in plasma cells and therefore well within the detection limits of non-radiolabelled biotin-streptavidin and similar detection systems, the development of non-radiolabelled κ-chain cRNA hybridisation probes suitable for bulk synthesis and therefore routine use would not appear to be an insuperable objective. The use of defined hybridisation probes also should eliminate much conflicting evidence obtained from similar clinical studies obtained using antisera of differing specificity, and in addition, overcome problems associated with the use of monoclonal antibodies on formalin fixed paraffin sections.

A larger more detailed study to determine whether plasma cell infiltration is a true independent prognostic factor or whether it is related to other histological features of prognosis in mammary tumours would now be of value. Such a study should include for comparison a parallel analysis of B and T cell markers using a panel of monoclonal antibodies.

We thank the Cancer Research Campaign for supporting the bulk of this work and Dr Philip Roberts for supplying the archival material. Collection of archival material and control tissue was supported by a grant from the East Anglian Regional Health Authority. We would also like to thank Neil Rayment and Lucinda Johns for valuable instruction and assistance in section preparation.

### References

AN, T., SOOD, U., PIETRUCK, T., CUMMINGS, G., HASHIMOTO, K. & CRISSMAN, J.D. (1987). *In situ* quantitation of inflammatory mononuclear cells in ductal infiltrating breast carcinoma. *Am. J. Pathol.*, **128**, 52.

BENTON, W.D. & DAVIES, R.W. (1977). Screening λgt recombinant clones by hybridisation to single plaques *in situ*. *Science*, **196**, 180.

BERK, A.J. & SHARP, P.A. (1977). Sizing and mapping of early adenovirus mRNAs by gel electrophoresis of $S_1$ endonuclease-digested hybrids. *Cell*, **12**, 721.

BLACK, M.M., BARCLAY, T.H.C. & HANKEY, B.F. (1975). Prognosis in breast cancer utilizing histological characteristics of the primary tumour. *Cancer*, **36**, 2048.

BLOOM, H.J.G. & RICHARDSON, W.W. (1957). Histological grading and prognosis in breast cancer. *Br. J. Cancer*, **11**, 359.

CHIRGWIN, J.M., PRZYBYLA, A.E., MACDONALD, R.J. & RUTTER, W.J. (1979). Isolation of biologically active ribonucleic acid from sources enriched in ribonucleases. *Biochemistry*, **18**, 5294.

COLLINS, M.K.L., KISSONERGHIS, A.M., DUNNE, M.J., WATSON, C.J., RIGBY, P.W.J. & OWEN, M.J. (1985). Transcripts from an aberrantly re-arranged human T cell receptor β-chain gene. *EMBO J.*, **4**, 1211.

COLLIS, P.J. (1988). Differential gene expression in human breast cancer. Ph.D. Thesis, University of London.

CRAIG, R.K., BROWN, P.A., HARRISON, O.S., McILREAVY, D. & CAMPBELL, P.N. (1976). Guinea-pig milk protein synthesis: Isolation and characterisation of mRNAs from lactating mammary gland and identification of caseins and pre-α-lactalbumin as translation products in heterologous cell-free systems. *Biochem. J.*, **160**, 57.

DAWSON, P.J., FERGUSON, D.J. & KARRISON, T. (1982). The pathologic findings of breast cancer in patients surviving 25 years after radical mastectomy. *Cancer*, **50**, 2131.

EDBROKE, M.R., PARKER, D., McVEY, J.H. & 4 others (1985). Expression of the human calcitonin/CGRP gene in lung and thyroid carcinoma. *EMBO J.*, **4**, 715.

FISHER, E.R., KOTWAL, N., HERMANN, C. & FISHER, B. (1983). Types of tumour lymphoid response and sinus histiocytosis. *Arch. Pathol. Lab. Med.*, **107**, 222.

HEITER, P.A., MAIZEL, J.V. & LEDER, P. (1982). Evolution of human immunoglobulin $K_J$ region genes. *J. Biol. Chem.*, **257**, 1516.

HOEFLER, H., CHILDERS, H., MONTMING, M.R., LECHAN, R.M., GOODMAN, R.H. & WOLFE, H.J. (1986). *In situ* hybridisation methods for the detection of somatostatin mRNA in tissue sections using antisense RNA probes. *Histochem. J.*, **18**, 597.

HOWELL, A., BARNES, D.M. & HARLAND, R.N.L. (1984). Steroid hormone receptors and survival after first relapse in breast cancer. *Lancet*, **i**, 558.

HSU, S.M., RAINE, L. & NAYAK, R.N. (1981). Medullary carcinoma of breast: an immunohistochemical study of its lymphoid stroma. *Cancer*, **48**, 1368.

HUANG, W.M., GIBSON, S.J., FACER, P., GU, J. & POLAK, J.M. (1983). Improved section adhesion for immunocytochemistry using high molecular wright polymers of L-lysine as a slide coating. *Histochem.*, **77**, 275.

HURLIMANN, J. & SARAGA, P. (1985). Mononuclear cells infiltrating human mammary carcinomas: immunohistochemical analysis with monoclonal antibodies. *Int. J. Cancer*, **35**, 753.

HUYNH, T.V., YOUNG, R.A. & DAVIS, R.W. (1985). Construction and screening of cDNA libraries in λgt10 and λgt11. In *DNA Cloning*, Vol. 1, Glover, D.M. (ed) p. 49, I.R.L. Press: Oxford.

KELLEY, D.E. & PERRY, R.P. (1986). Transcriptional and post-transcriptional control of immunoglobulin mRNA production during B lymphocyte development. *Nucleic Acids Res.*, **14**, 5431.

KLOBECK, H.G., BORNKAMM, G.W., COMBRIATO, G., MOCIKAT, R., POHLENZ, H.-D. & ZACHAU, H.G. (1985). Sub-group IV of human immunoglobulin κ light chains is encoded by a single germline gene. *Nucleic Acids Res.*, **18**, 6515.

LAUDER, I., AHERNE, W., STEWART, J. & SAINSBURY, R. (1977). Macrophage infiltration of breast tumours: a prospective study. *J. Clin. Pathol.*, **30**, 563.

LEATHAM, A.J. & BROOKS, S.A. (1987). Predictive value of lectin binding on breast cancer recurrence and survival. *Lancet*, **i**, 1054.

LWIN, K.Y., ZUCCARINI, O., SLOANE, J.P. & BEVERLEY, P.C.L. (1985). An immunohistological study of leucocyte localisation in benign and malignant breast disease. *Int. J. Cancer*, **36**, 433.

MANIATIS, T., FRITSCH, E.F. & SAMBROOK, J. (1982). *Molecular Cloning: A Laboratory Manual.* Cold Spring Harbour Laboratory Press: Cold Spring Harbour, New York.

MARSH, P., MILLS, F. & GOULD, H. (1985). Detection of a unique $V_{K_{IV}}$ germline gene by a cloned cDNA probe. *Nucleic Acids Res.*, **18**, 6531.

MELTON, D.A., KRIEG, P.A., REBAGLIATI, M.R., MANIATIS, T., ZINN, K. & GREEN, M.R. (1984). Efficient *in vitro* synthesis of biologically active RNA and RNA hybridisation probes from plasmids containing a bateriophage SP6 promoter. *Nucleic Acids Res.*, **12**, 7035.

MYERS, R.M., LUMELSKY, N., LERMAN, L.S. & MANIATIS, T. (1985). Detection of single base substitutions in total genomic DNA. *Nature*, **313**, 495.

PEMBLE, S.E., TAYLOR, J.B. & KETTERER, B. (1986). Tissue distribution of rat glutathione transferase subunit 7, a hepatoma marker. *Biochem. J.*, **240**, 885.

RIDOLPHI, R.L., ROSEN, P.P., PORT, A., KINNE, D. & MIKE, V. (1977). Medullary carcinoma of the breast. A clinipathologic study with 10 year follow-up. *Cancer*, **40**, 1365.

RIGBY, P.W.J., DIECKMANN, M., RHODES, C. & BERG, P. (1977). Labelling deoxyribonucleic acid to a high specific activity *in vitro* by nick-translation with DNA polymerase I. *J. Mol. Biol.*, **113**, 237.

RILEY, J.H., EDBROOKE, M.R. & CRAIG, R.K. (1986). Ectopic synthesis of high $M_r$ calcitonin by the BEN lung carcinoma cell-line reflects aberrant proteolytic processing. *FEBS. Lett.*, **198**, 71.

ROSES, D.F., BELL, D.A., FLOTTE, T.J., TAYLOR, R., RATECH, H. & DUBIN, N. (1982). Pathogenic predictors of recurrence in stage 1 (TINOMO) breast cancer. *Am. J. Clin. Pathol.*, **78**, 817.

SANGER, F., NICKLEN, S. & COULSON, A.R. (1977). DNA sequencing with chain termination inhibitors. *Proc. Natl Acad. Sci. USA*, **74**, 5463.

SCHOORL, R., BRUTEL DE LA RIVIERE, A., KR. VON DERN BORNE. A.E.G. & FELTKAMPVROOM, T.M. (1976). Identification of T and B lymphocytes in human breast with immunohistochemical techniques. *Am. J. Pathol.*, **84**, 529.

SLAMON, D.J., CLARK, G.M., WONG, S.G., LEVIN, W.J., ULLRICH, A. & McGUIRE. W.L. (1987). Human breast cancer: correlation of relapse and survival with amplification of the HER-2/neu oncogene. *Science*, **235**, 177.

STENKVIST, B., BENGTSSON, E., DAHLQVIST, B. & 4 others (1982). Predicting breast cancer recurrence. *Cancer*, **50**, 2884.

TAYLOR, J.B., CRAIG, R.K., BEALE, D. & KETTERER, B. (1984). Construction and characterisation of a plasmid containing complementary DNA to mRNA encoding the N-terminal amino acid sequence of the rat glutathione Ya subunit. *Biochem. J.*, **219**, 223.

UNDERWOOD, J.C.E. (1974). Lymphoreticular infiltration in human tumours: Prognostic and biological implications: a review. *Br. J. Cancer*, **30**, 538.

VON KLEIST, S., BERLING, J., BOHLE, W. & WITTERKIND, CH. (1987). Immunohistological analysis of lymphocyte subpopulations infiltrating breast carcinomas and benign lesions. *Int. J. Cancer*, **40**, 18.

WEIGERT, M., PERRY, R., KELLY, D., HUNKAPILLER, T., SCHILLING, J. & HOOD, L. (1980). The joining of V and J gene segments create antibody density. *Nature*, **283**, 497.

WILKINSON, M.J.S., HOWELL, A., HARRIS, M., TAYLOR-PAPADIMITRIOU, J., SWINDELL, R. & SELLWOOD, R.A. (1984). The prognostic significance of two epithelial membrane antigens expressed by human mammary carcinomas. *Int. J. Cancer*, **33**, 299.

ZUK, J.A. & WALKER, R.A. (1987). Immunohistochemical analysis of HLA antigens and mononuclear infiltrates of benign and malignant breast. *J. Pathology*, **152**, 275.